# The Safety of Apixaban Compared to Warfarin in Hospitalized Patients with Acute Kidney Injury

**DOI:** 10.3390/jcm14134685

**Published:** 2025-07-02

**Authors:** Majed S. Al Yami, Lama Alfehaid, Abdulmajeed M. Alshehri, Norah Alqahtani, Ghadah Almuaither, Shaden H. Alonazi, Mohammed Y. Alzahrani, Amal M. Badawoud, Omar A. Almohammed

**Affiliations:** 1 Department of Pharmacy Practice, College of Pharmacy, King Saud Bin Abdulaziz University for Health Sciences, 2915 Al Haras Al Watani St, Ar Rimayah, Riyadh 14611, Saudi Arabia; fehaidl@ksau-hs.edu.sa (L.A.); shehriabdul@ksau-hs.edu.sa (A.M.A.); alqahtani0330@ksau-hs.edu.sa (N.A.); almuaither337@ksau-hs.edu.sa (G.A.); alonazi131@ksau-hs.edu.sa (S.H.A.); alzahranimoham@ksau-hs.edu.sa (M.Y.A.); 2 Pharmaceutical Care Department, King Abdulaziz Medical City, Riyadh 11426, Saudi Arabia; 3 King Abdullah International Medical Research Center, Riyadh 11481, Saudi Arabia; 4 Department of Pharmacy Practice, College of Pharmacy, Princess Nourah Bint Abdulrahman University, P.O. Box 84428, Riyadh 11671, Saudi Arabia; ambadawoud@pnu.edu.sa; 5 Department of Clinical Pharmacy, College of Pharmacy, King Saud University, Riyadh 11451, Saudi Arabia; oalmohammed@ksu.edu.sa

**Keywords:** apixaban, warfarin, acute kidney injury, bleeding, thromboembolisms

## Abstract

**Background/Objectives**: Apixaban is favored over warfarin for atrial fibrillation (Afib) and venous thromboembolism (VTE) due to its effectiveness, safety, and lack of routine monitoring. However, managing anticoagulation in hospitalized patients with acute kidney injury (AKI) is challenging due to altered pharmacokinetics and limited safety data. This study assesses the safety and efficacy of apixaban versus warfarin in these patients. **Methods**: This retrospective chart review at King Abdulaziz Medical City in Riyadh included adult patients (≥18 years) with AKI, as defined by the Kidney Disease Improving Global Outcome (KDIGO) guideline. Primary outcomes were rates of major and minor bleeding within 30 days, as defined by the International Society on Thrombosis and Haemostasis (ISTH), and thrombotic events. Secondary outcomes included 30-day rates of all-cause mortality and hospital readmissions. **Results**: Among 513 patients, 294 received apixaban and 219 received warfarin. Major bleeding within 30 days was significantly lower in the apixaban group (3.4%) compared to warfarin (7.3%) (*p* = 0.0461). Minor bleeding rates were similar (6.5% apixaban vs. 5.5% warfarin; *p* = 0.616). Thrombotic events occurred in 6.8% of patients, with no significant difference between apixaban (6.5%) and warfarin (7.3%) (*p* = 0.739). Mortality rates were 8.0%, with no significant difference (8.8% apixaban vs. 6.8% warfarin; *p* = 0.3846). Readmission rates were comparable (38.8% for apixaban vs. 39.7% for warfarin; *p* = 0.9499). **Conclusions**: In hospitalized AKI patients, apixaban was associated with a lower major bleeding risk compared to warfarin, with similar rates of thrombotic events, mortality, and readmissions, suggesting apixaban may be a safer option, warranting further research.

## 1. Introduction

Direct oral anticoagulants (DOACs) have replaced warfarin in many indications due to similar efficacy, a better safety profile, fewer food and drug interactions, and the lack of the need for continuous coagulation monitoring. Warfarin may still be preferred in specific indications, such as patients with atrial fibrillation (Afib) with moderate to severe mitral stenosis, with mechanical heart valves, or with rheumatic heart diseases [1]. While warfarin has a lower initial cost than DOACs, the additional expenses for regular blood tests and INR monitoring can increase its overall cost. Conversely, apixaban has been shown to be more cost-effective than warfarin, with incremental cost-effectiveness ratios per event-free life year gained of USD 10,501, USD 7809, and USD 758, respectively [2]. The 2023 American College of Cardiology/American Heart Association/American College of Clinical Pharmacy/Heart Rhythm Society (ACC/AHA/ACCP/HRS) Guidelines for the Diagnosis and Management of Atrial Fibrillation recommend using DOAC as first-line agents for patients with Afib with moderate to severe mitral stenosis or mechanical heart valves [1].

Among DOACs, apixaban is frequently used due to its well-established efficacy and safety profile, as well as its favorable pharmacokinetic profile, which makes it a reasonable choice in high-risk patients, including those with renal impairment [3,4,5,6,7]. Apixaban has a quick onset of action and can reach its peak plasma concentration within 1 to 4 h after oral administration. It has a half-life of 12 h, which justifies its twice-a-day dosing with predictable metabolism and clearance. In contrast to other DOACs, apixaban renal excretion accounts for only 27% of its elimination, while the remainder is metabolized by the liver via the CYP3A4 enzyme, which makes it a reasonable choice for patients with mild to moderate renal impairment with creatinine clearance (CrCl) of ≥25 mL/min [8]. Evidence is scarce regarding apixaban use in patients with severe renal impairment (CrCl < 25 mL/min) who are not yet on dialysis, where the pharmacokinetic profile of the medication is significantly altered, raising safety concerns for bleeding, as the majority of this population was excluded from the landmark clinical trials [3,4,9].

The management of anticoagulants presents an increasingly complex issue in the context of acute kidney injury (AKI), which affects approximately 15% of hospitalized patients [10], due to the scarcity of robust evidence regarding the safety and efficacy of apixaban or warfarin in this population. In clinical practice, some practitioners opt to withhold oral anticoagulants during hospitalization when the risk of thrombosis is deemed low, while others may choose to transition to a parenteral anticoagulant based on the assessed risk of thrombosis or stroke. There remains a notable absence of standardized practices concerning the administration of oral anticoagulants in patients with AKI, attributable to the insufficient evidence and underrepresentation in major randomized controlled trials [3,4]. This study aimed to address the knowledge gap by comparing the safety and efficacy of apixaban and warfarin in hospitalized patients presenting with AKI.

## 2. Materials and Methods

### 2.1. Study Site and Design

This retrospective cohort study was conducted at King Abdulaziz Medical City (KAMC), part of the Ministry of National Guard Health Affairs (MNGHA) in Riyadh, Saudi Arabia, from 1 January 2017 to 31 October 2023. The study protocol received approval from the Institutional Review Board (IRB) of King Abdullah International Medical Research Center (KAIMRC) at MNGHA (IRB Approval No.: SP23R/206/09). KIMARC IRB waived patient consent due to the retrospective nature of the study. There was no direct interaction with patients or collection of biological samples. Instead, the study relied on the review of existing patient records. Data was extracted only after obtaining ethical approval from the institutional review board of KAIMRC. The study was conducted in accordance with the World Medical Association Declaration of Helsinki—Ethical Principles for Medical Research Involving Human Subjects (adopted 1964; updated 2013) and the Strengthening of the Reporting of Observational Studies in Epidemiology (STORB) guideline (Supplementary Materials).

### 2.2. Study Subjects

This study included all adult patients aged ≥18 years old who were on either apixaban or warfarin for venous thromboembolism (VTE) or Afib with AKI (as defined by the Kidney Disease: Improving Global Outcome (KDIGO) guideline; rise in serum creatinine (SCr) ≥ 0.3 mg/dL within 48 h; rise in SCr ≥ 1.5 times baseline, which is known or presumed to have occurred within the prior seven days; or urine output < 0.5 mL/kg/hour for six hours) [11]. The eligible patients were categorized into two groups based on their pre-admission use of anticoagulants: apixaban or warfarin. Patients were excluded if they were taking warfarin and had an International Normalized Ratio (INR) outside the target range (2–3), had received parenteral anticoagulants within 72 h of starting oral anticoagulants, switched to parenteral anticoagulants at any time after screening, had a diagnosis of ESRD, or were undergoing hemodialysis.

### 2.3. Study Outcomes

The primary outcomes of the study were major bleeding, as defined by the International Society on Thrombosis and Haemostasis (ISTH): “any fatal bleeding, and/or symptomatic bleeding in a critical area or organ, such as intracranial, intraspinal, intraocular, retroperitoneal, intra-articular, pericardial, or intramuscular with compartment syndrome, and/or bleeding causing a fall in hemoglobin level of 20 g/L (1.24 mmol/L) or more or leading to the transfusion of two or more units of whole blood or red cells,” and minor bleeding, defined as any bleeding that does not meet the criteria for major bleeding [12]. Additional outcomes included the need for blood transfusion and the incidence of thrombotic events (deep vein thrombosis (DVT), pulmonary embolism (PE), or ischemic stroke) during hospitalization and within 30 days post-discharge. The secondary outcomes were the rate of hospital readmission and all-cause mortality during hospitalization and within 30 days post-discharge. Study variables, including age, gender, comorbidities, indication for anticoagulation, AKI stage, presence of drug–drug interactions, CHA_2_DS_2_-VASc Score, HAS-BLED Score, and laboratory findings, were collected retrospectively.

### 2.4. Statistical Analysis

Continuous variables were summarized as means (standard deviations) and compared using the independent *t*-test, while categorical variables were presented as frequencies and percentages and analyzed with either Pearson’s chi-square test or Fisher’s exact test. Two subgroup analyses were performed based on anticoagulation indication and AKI stage (based on KDIGO criteria) [13] to assess differences in the outcomes. Furthermore, a backward-stepwise multivariable logistic regression model was performed to evaluate the impact of patient characteristics and past medical history on major bleeding, thrombotic events, and mortality. The following variables were included in the regression: age, medication (warfarin or apixaban), gender, comorbidities (hypertension, diabetes mellitus, chronic kidney disease, ischemic heart disease, respiratory disease, hypothyroidism, liver disease, stroke), indications for anticoagulation, CHADS-VASc Score, and HAS-BLED Score. All data were analyzed using Stata/SE statistical software Version 15.1 (StataCorp LLC).

## 3. Results

### 3.1. Baseline Characteristics

**Figure 1:**
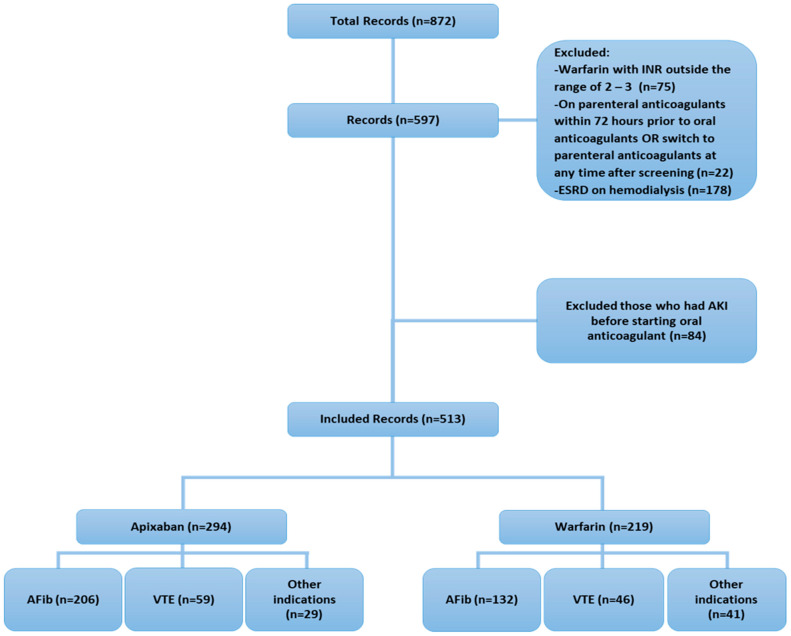
Study flowchart.

Throughout the study period, 513 patients met our inclusion criteria; Figure 1 illustrates the study flowchart and the number of screened, included, and excluded patients. The baseline demographic characteristics indicate an average age of 73.4 ± 13.9; apixaban patients were statistically older compared to the warfarin group, at 75 ± 13.0 and 71.3 ± 14.7, respectively (*p* = 0.0028). The sex distribution was comparable between groups. Among comorbidities, the apixaban group had a higher proportion of diabetic patients compared to the warfarin group, 223 (75.9%) vs. 149 (68%) (*p* = 0.0499). In addition to that, dyslipidemia and ischemic heart disease were higher in the apixaban group compared to the warfarin group (101 (34.4%) vs. 51 (23.3%) (*p* = 0.0066)) and (85 (28.9%) vs. 39 (17.8%) (*p* = 0.0037)), respectively. Other comorbidities were comparable between groups. Chronic kidney disease (CKD) was found in 174 (33.9%), which was comparable between both groups. Almost 67% of cases were anticoagulated for atrial fibrillation, whereas 21.3% received either apixaban or warfarin for venous thromboembolism (*p* = 0.0060). The drug–drug interactions between apixaban or warfarin and other home medications were assessed, and the distribution of those interactions was comparable between groups. The baseline bleeding risk score (HAS-BLED) was comparable between apixaban and warfarin. However, the thromboembolic risk score (CHADVASC2) was higher in apixaban compared to warfarin (4.8 ± 1.4 vs. 4.3 ± 1.4 (*p* = 0.0150)). The baseline SCr prior to admission and during admission was comparable between groups. Additionally, among patients who received a blood transfusion, the hemoglobin level prior to transfusion was comparable between groups. Lastly, the AKI stage or SCr fold changes from baseline were comparable between both groups (Table 1).

### 3.2. Clinical Outcomes

Major bleeding was more prevalent among warfarin patients compared to those on apixaban (10 (3.4%) vs. 16 (7.3%) (*p* = 0.0461)). In contrast, the rates of minor bleeding and the need for blood transfusions were comparable between the groups (19 (6.5%) vs. 12 (5.5%) (*p* = 0.6439) and 24 (8.2%) vs. 24 (11.0%) (*p* = 0.2822), respectively. Thrombotic events developed in 35 (6.82%) patients and were comparable between groups, including DVT, PE, or stroke, with no statistical difference (*p* = 0.7079). Out of the developed thrombotic events, the most common type was DVT 14 (40%), followed by stroke (28.6%), other thrombotic events 6 (17.1%), and PE 5 (14.3%) (Table 2).

### 3.3. Subgroup Analysis of Clinical Outcomes

In the subgroup analysis, thrombotic events were higher in patients who received anticoagulation for managing VTE compared to those who are anticoagulated for Afib, 18 (10.4%) compared to 17 (5%) (*p* = 0.0217). None of the other outcomes showed a distinct difference between indications. In addition to that, when outcomes were sub-analyzed based on AKI stage, major bleeding and blood transfusion were higher in stage 2 or 3 compared to stage 1 (16 (9.4%) vs. 10 (3.1%) (*p* = 0.0025)) and (25 (14.7%) vs. 23 (7.0%) (*p* = 0.0060)), respectively. Other outcomes were comparable between AKI stages (Table 3).

### 3.4. Multivariable Analysis of Factors Associated with Clinical Outcomes

When multivariable analysis was conducted to assess factors associated with major bleeding, apixaban was associated with a statistically significant lower risk of bleeding at 58% compared to the warfarin group (OR 0.42, 95% CI: 0.18–0.96). No other factors demonstrated significant risks (Table 4).

Thrombotic events were four times higher in patients who were anticoagulated for DVT compared to those who were not (OR 4.75, 95% CI: 1.82–12.4). Furthermore, having a past medical history of hypertension, respiratory diseases, or liver disease showed more thrombosis events with no statistical significance (OR 2.21, 95% CI: 0.72–6.76), (OR 2.14, 95% CI: 0.89–5.13) and (OR 2.92, 95% CI: 1.01–8.41), respectively.

Compared to patients with atrial fibrillation, those with DVT had a significantly increased risk of experiencing a thrombotic event (OR: 4.75, 95% CI: 1.82–12.4), indicating that they were approximately 4.75 times more likely to develop a thrombotic event. Similarly, patients with pulmonary embolism showed an elevated but non-significant risk (OR: 1.74, 95% CI: 0.55–5.48). Additionally, patients with other indications had a higher but non-significant risk of thrombotic events compared to the atrial fibrillation group (OR: 2.35, 95% CI: 0.86–6.47) (Table 4).

Regarding mortality risk and comorbidities, patients with heart failure had a twofold increased likelihood of death compared to those without heart failure (OR: 2.08, 95% CI: 1.04–4.13). Stroke and hypothyroidism were also associated with an elevated risk of mortality, though not statistically significant (OR: 2.43, 95% CI: 0.86–6.91) and (OR: 1.79, 95% CI: 0.77–4.16), respectively. Chronic kidney disease appeared to be associated with a lower risk of mortality (OR: 0.61, 95% CI: 0.29–1.27), though this was also not significant (Table 4).

Patients with DVT (OR: 2.20, 95% CI: 0.81–5.95) and PE (OR: 2.33, 95% CI: 0.93–5.85) had an increased risk of mortality compared to those with atrial fibrillation, though these associations did not reach statistical significance. No deaths were observed in patients who had DVT and PE. Patients with other indications had a lower mortality risk (OR: 0.44, 95% CI: 0.10–1.95), though this was not statistically significant (Table 4).

## 4. Discussion

In this real-world study, we aimed to evaluate the safety and efficacy of apixaban compared to warfarin in hospitalized patients with AKI. Practitioners face significant concerns about continuing apixaban in these patients due to the unpredictability of drug clearance, risk of drug accumulation, and potential for bleeding complications. The inconsistency in practice and lack of clear guidelines further complicate clinical decisions regarding whether to continue oral anticoagulants when AKI develops or to switch to parenteral short-acting heparin therapy. Given the persistent ambiguity in this area, largely due to insufficient data and its underrepresentation in landmark randomized controlled trials, our study addresses a significant gap in clinical knowledge and provides insights for better patient management.

The results of our study revealed that patients receiving warfarin had a significantly higher incidence of major bleeding compared to those on apixaban. There was no difference in the incidence of minor bleeding, the need for blood transfusion, 30-day re-hospitalization, or mortality among patients on warfarin compared to those on apixaban. Supporting studies evaluating the safety of the continuation of apixaban during AKI, specifically, are limited. Givens et al. investigated bleeding rates among 232 patients using apixaban, both with and without AKI. In the AKI group, there was a reported incidence of major bleeding associated with apixaban that was higher than typically seen; however, the rates for major and minor bleeding did not reach statistical significance (AKI group 7.8% vs. non-AKI 3.4%; *p* = 0.2). The regression analysis indicated that the concurrent use of a P2Y12 inhibitor was a predictor of major bleeding [13]. In our study, we examined the presence of drug–drug interactions and assessed the baseline risk of bleeding using the widely utilized bleeding prediction tool, HAS-BLED, along with all other baseline characteristics to predict the odds ratio in a backward-stepwise multivariable logistic regression model. The results showed 58% lower odds of major bleeding compared to warfarin.

Several potential mechanisms may explain the favorable bleeding profile of apixaban in patients with AKI compared to warfarin. As a direct factor Xa inhibitor, apixaban provides a more predictable anticoagulant effect, significantly reducing the risk of major bleeding, as demonstrated in landmark trials [3,4] and proven in existing literature [5,6,7]. Despite prolonged elimination in AKI, its shorter half-life allows for quicker reversal of effects, mitigating bleeding complications. Its stable pharmacokinetics and minimal food interactions further decrease bleeding risks. Additionally, with only 27% renal excretion, apixaban is well-suited for patients with mild to moderate kidney impairment, which is particularly relevant in AKI. Together, these factors may explain the observed outcomes.

Moreover, subgroup analysis of the patients included revealed a heightened risk of major bleeding and an increased need for blood transfusions among those with advanced-stage AKI (KDIGO AKI stages 2 or 3). These findings are supported by a single-center study, which demonstrated that patients with advanced AKI were more likely to experience bleeding incidents, regardless of whether they were receiving anticoagulant therapy [14]. This suggests that the severity of kidney impairment itself significantly contributes to bleeding risk, highlighting the need for careful management of such patients. Furthermore, multivariate analysis demonstrated a significantly higher risk of recurrent thromboembolic events among patients with VTE compared to those with Afib, highlighting the critical need for adequate apixaban plasma drug levels and corresponding anti-factor Xa activity. This heightened risk in VTE, coupled with the distinct pathophysiology of venous versus arterial thrombus formation, suggests that empiric dose reduction in apixaban in VTE patients with AKI should be avoided [15,16,17]. The standard apixaban dosing regimen for VTE treatment was evaluated in the AMPLIFY trial for patients with adequate renal function (CrCl ≥ 25 mL/min) [4]. While the FDA-approved labeling indicates no renal dose adjustment is necessary for apixaban in VTE, even in ESRD [18], it is important to note that clinical trials in these specific populations are limited. Therefore, careful management of apixaban dosing in VTE patients with AKI is crucial to prevent further thromboembolic complications, ensuring therapeutic drug levels without increasing bleeding risk, particularly considering the higher background risk of recurrence in VTE compared to Afib. Routine monitoring is not required; however, an anti-Xa assay may be useful in specific situations, such as renal insufficiency.

Overall, this study highlights the safety and efficacy of apixaban in hospitalized patients with AKI, suggesting that holding oral anticoagulants or transitioning to short-term heparin infusion may not be necessary. For patients with a history of recurrent AKI and unstable renal function, apixaban may be a safer alternative, except in cases where there are compelling indications for warfarin, such as the presence of mechanical heart valves, moderate to severe mitral stenosis, or significant rheumatic heart disease. It is important to avoid reducing the dose of apixaban in patients with VTE and concurrent AKI, as this could increase the risk of recurrent thrombosis during hospitalization or within 30 days post-discharge.

To the best of our knowledge, this study is the first to investigate the safety of apixaban compared to warfarin in patients with AKI within a real-world context. The study included a substantial sample size and considered several potential confounders in its analysis. Nonetheless, several limitations must be acknowledged. Firstly, the study was conducted at a single center, which may restrict the generalizability of the findings to other settings or populations. Secondly, patients requiring temporary hemodialysis were excluded, limiting the applicability of the results to this subgroup. However, existing evidence supports the safety of apixaban use in patients undergoing hemodialysis [19,20]. Another limitation is the 30-day follow-up period, which we chose to evaluate the immediate safety and effectiveness of anticoagulation in AKI patients. This timeframe enables us to assess early bleeding and thrombotic events, but it may not capture long-term outcomes, especially in patients recovering from AKI or developing CKD. Longer follow-up studies are needed for a more comprehensive understanding. Furthermore, our retrospective design relies on existing patient records, which might include incomplete or missing data, potentially affecting accuracy, such as the actual apixaban dose administered. The lack of randomization could introduce inherent differences between groups that influence outcomes. Although we adjusted for multiple confounders, unmeasured variables might still impact results. Future multicenter randomized controlled trials are essential to validate these findings.

## 5. Conclusions

This study provides valuable insights into the safety and efficacy of apixaban compared to warfarin in hospitalized patients with AKI. The findings indicate that apixaban was associated with a significantly lower risk of major bleeding compared to warfarin while maintaining similar rates of thrombotic events, mortality, and hospital readmissions. These results suggest that apixaban may serve as a safer alternative to warfarin for anticoagulation in a patient population characterized by unstable renal function or recurrent AKI, especially considering the challenges of managing anticoagulation in the context of AKI. Nonetheless, the retrospective design and single-center nature of this study may limit the generalizability of the findings. Further research, including prospective and multicenter studies, is essential to confirm these findings and to establish comprehensive clinical guidelines for managing anticoagulation in patients with AKI.

## Figures and Tables

**Table 1:** Baselines characteristics.

Characteristics	Overall (n = 513)	Apixaban (n = 294)	Warfarin (n = 219)	*p*-Value
Demographics				
Age	73.4 ± 13.9	75 ± 13.0	71.3 ± 14.7	**0.0028**
Gender				0.4584
Male	251 (48.9)	148 (50.3)	103 (47.0)	
Female	262 (51.1)	146 (49.7)	116 (53.0)	
Comorbidities *				
Hypertension	427 (83.2)	248 (84.4)	179 (81.7)	0.4323
Diabetes Miletus	372 (72.5)	223 (75.9)	149 (68.0)	**0.0499**
Heart Failure	195 (38.0)	117 (39.8)	78 (35.6)	0.3347
Chronic Kidney Disease	177 (34.5)	94 (32.0)	83 (37.9)	0.1625
Dyslipidemia	152 (29.6)	101 (34.4)	51 (23.3)	**0.0066**
Ischemic Heart Disease	124 (24.2)	85 (28.9)	39 (17.8)	**0.0037**
Respiratory Disease	80 (15.6)	46 (15.6)	34 (15.5)	0.9702
Hypothyroidism	72 (14.0)	38 (12.9)	34 (15.5)	0.4017
Liver Disease	55 (10.7)	29 (9.9)	26 (11.9)	0.4671
Stroke	33 (6.4)	20 (6.8)	13 (5.9)	0.6923
Indication				**0.0060**
Atrial Fibrillation	340 (66.3)	208 (70.7)	132 (60.3)	
DVT	54 (10.5)	27 (9.2)	27 (12.3)	
PE	49 (9.6)	31 (10.5)	18 (8.2)	
DVT+PE	6 (1.2)	4 (1.4)	2 (0.9)	
Other	64 (12.5)	24 (8.2)	40 (18.3)	
Drug–Drug interaction	199 (38.8)	116 (39.5)	83 (37.9)	0.7205
Drug–Drug interaction category				
Strong	10 (1.9)	7 (2.4)	3 (1.4)	0.7311
Moderate	162 (31.6)	93 (31.6)	69 (31.5)	
Pharmacodynamic	27 (5.3)	16 (5.4)	11 (5.0)	
Thromboembolic and Bleeding Risk				
Chadsvasc Score	4.6 ± 1.4	4.8 ± 1.4	4.3 ± 1.4	**0.0150**
HAS-BLED Score	2.7 ± 1.0	2.7 ± 1.0	2.8 ± 1.1	0.9292
Laboratory values				
Baseline SCr,	1.7 ± 1.2	1.7 ± 1.2	1.8 ± 1.2	0.3837
SCr admission	3.2 ± 2.2	3.1 ± 2.1	3.5 ± 2.5	0.1052
Pre-transfusion HgB **	74.3 ± 21.7	74.6 ± 21.7	74.1 ± 22.4	0.6152
AKI Stages ***				0.2351
Stage 1	327 (63.7)	189 (64.3)	138 (63.0)	
Stage 2	104 (20.3)	61 (20.7)	43 (19.6)	
Stage 3	66 (12.9)	32 (10.9)	34 (15.5)	
Not documented	16 (3.1)	12 (4.1)	4 (1.8)	
SCr fold changes from baseline	2.1 ± 1.5	2.1 ± 1.4	2.2 ± 1.5	0.2976

* only comorbidities that affected more than 5% of the patients were listed here; ** this is only for patients who needed blood transfusion. *** Based on KDIGO criteria for AKI stages.

**Table 2:** Outcomes during admission or within 30 days of discharge.

	Overall (n = 513)	Apixaban (n = 294)	Warfarin (n = 219)	*p*-Value
Major bleeding	26 (5.1)	10 (3.4)	16 (7.3)	**0.0461**
Minor bleeding	31 (6.0)	19 (6.5)	12 (5.5)	0.6439
Blood transfusion	48 (9.4)	24 (8.2)	24 (11.0)	0.2822
Thrombotic event	35 (6.8)	19 (6.5)	16 (7.3)	0.7079
Type of thrombotic event				0.4728
DVT	14 (2.7)	8 (2.7)	6 (2.7)	
PE	5 (1.0)	4 (1.4)	1 (0.5)	
Stroke	10 (1.9)	5 (1.7)	5 (2.3)	
Other	6 (1.2)	2 (0.7)	4 (1.8)	
Readmission or ED visit	202 (39.4)	115 (39.1)	87 (39.7)	0.8887
Death	41 (8.0)	26 (8.8)	15 (6.8)	0.4100

**Table 3:** Subgroup analysis for the clinical outcomes based on indication and AKI stage.

Outcomes	Indication			AKI Stage *		
	Atrial Fibrillation (n = 340)	Other Indications (VTE and Others) (n = 173)	*p*-Value ^†^	Stage 1 (n = 327)	Stages 2 or 3 (n = 170)	*p*-Value ^†^
Major bleeding	13 (3.8)	13 (7.5)	0.0716	10 (3.1)	16 (9.4)	**0.0025**
Minor bleeding	23 (6.8)	8 (4.6)	0.3361	17 (5.2)	13(7.7)	0.2769
Blood transfusion	29 (8.5)	19 (11.0)	0.3670	23 (7.0)	25 (14.7)	**0.0060**
Thrombotic event	17 (5.0)	18 (10.4)	**0.0217**	23 (7.0)	10 (5.9)	0.6248
Readmission or ED visit	135 (39.7)	67 (38.7)	0.8304	126 (38.5)	73 (42.9)	0.3413
Death	26 (7.7)	15 (8.7)	0.6861	21 (6.4)	19 (11.2)	0.0645

Numbers are presented as frequency (%); * patients with no documented stage for AKI were excluded from the analysis. ^†^ *p*-values are from the chi-square test; values in bold are statistically significant. Abbreviation: AKI: acute kidney injury.

**Table 4:** Multivariable analysis to investigate factors associated with major bleeding, thrombotic events, and death.

Variables/Outcomes	Major Bleeding			Thrombotic Events			Mortality		
	No	Yes	OR (95% CI) *	No	Yes	OR (95% CI) *	No	Yes	OR (95% CI) *
Age	73.7 ± 13.8	69.5 ± 14.4	---	73.5 ± 13.9	72.7 ± 13.3	---	73.1 ± 14.0	77.9 ± 11.2	---
Medication, warfarin	203 (92.7)	16 (7.3)	Ref	203 (92.7)	16 (7.3)	---	204 (93.2)	15 (6.8)	---
Apixaban	284 (96.6)	10 (3.4)	**0.42 (0.18–0.96)**	275 (93.5)	19 (6.5)	---	268 (91.2)	26 (8.8)	---
Gender, female (vs. male)	247 (94.3)	15 (5.7)	---	243 (92.7)	19 (7.3)	---	239 (91.2)	23 (8.8)	---
Comorbidities									
Hypertension	407 (95.3)	20 (4.7)	---	396 (92.7)	31 (7.3)	2.21 (0.72–6.76)	393 (92.0)	34 (8.0)	---
Diabetes Miletus	352 (94.6)	20 (5.4)	---	348 (93.5)	24 (6.5)	---	342 (91.9)	30 (8.1)	---
Heart Failure	184 (94.4)	11 (5.6)	---	186 (95.4)	9 (4.6)	---	174 (89.2)	21 (10.8)	**2.08 (1.04–4.13)**
Chronic Kidney Disease	168 (94.9)	9 (5.1)	---	165 (93.2)	12 (6.8)	---	166 (93.8)	11 (6.2)	0.61 (0.29–1.27)
Dyslipidemia	142 (93.4)	10 (6.6)	1.77 (0.77–4.07)	139 (91.4)	13 (8.6)	---	136 (89.5)	16 (10.5)	---
Ischemic Heart Disease	117 (94.4)	7 (5.6)	---	117 (94.4)	7 (5.6)	---	114 (91.9)	10 (8.1)	---
Respiratory Disease	73 (91.3)	7 (8.8)	2.15 (0.86–5.34)	72 (90.0)	8 (10.0)	2.14 (0.89–5.13)	72 (90.0)	8 (10.0)	---
Hypothyroidism	69 (95.8)	3 (4.2)	---	67 (93.1)	5 (6.9)	---	64 (88.9)	8 (11.1)	1.79 (0.77–4.16)
Liver Disease	50 (90.9)	5 (9.1)	2.10 (0.75–5.91)	50 (90.9)	5 (9.1)	---	50 (90.9)	5 (9.1)	---
Stroke	32 (97.0)	1 (3.0)	---	28 (84.8)	5 (15.2)	2.92 (1.01–8.41)	28 (84.8)	5 (15.2)	2.43 (0.86–6.91)
Indication									
Atrial Fibrillation	327 (96.2)	13 (3.8)	---	323 (95.0)	17 (5.0)	Ref	314 (92.4)	26 (7.6)	Ref
Deep Vein Thrombosis (DVT)	50 (92.6)	4 (7.4)	---	46 (85.2)	8 (14.8)	**4.75 (1.82–12.4)**	48 (88.9)	6 (11.1)	2.20 (0.81–5.95)
Pulmonary Embolism (PE)	45 (91.8)	4 (8.2)	---	45 (91.8)	4 (8.2)	1.74 (0.55–5.48)	42 (85.7)	7 (14.3)	2.33 (0.93–5.85)
DVT + PE	6 (100.0)	0 (0.0)	---	6 (100.0)	0 (0.0)	NA	6 (100.0)	0 (0.0)	NA
Other	59 (92.2)	5 (7.8)	---	58 (90.6)	6 (9.4)	2.35 (0.86–6.47)	62 (96.9)	2 (3.1)	0.44 (0.10–1.95)
Thromboembolic and Bleeding Risk									
CHADS-VASc Score	4.6 ± 1.4	4.9 ± 1.7	---	4.6 ± 1.3	5.1 ± 2.1	---	4.6 ± 1.3	4.8 ± 1.7	---
HAS-BLED Score	2.7 ± 1.0	4 ± 0.9	---	2.7 ± 1.0	3.3 ± 1.1	---	2.7 ± 1.0	3.0 ± 1.2	---

Numbers are presented as mean ± SD or frequency (%); * ORs are the adjusted odds ratio from the backward-stepwise multivariable logistic regression model including patient characteristics and past medical history.

## Data Availability

The data that support the findings of this study are available from the corresponding author, but restrictions apply to the availability of these data.

## Supplementary Materials

The following supporting information can be downloaded at https://www.mdpi.com/article/10.3390/jcm14134685/s1, STROBE Statement—checklist.
